# Associations Between Health Literacy, eHealth Literacy, and COVID-19–Related Health Behaviors Among Chinese College Students: Cross-sectional Online Study

**DOI:** 10.2196/25600

**Published:** 2021-05-06

**Authors:** Shaojie Li, Guanghui Cui, Atipatsa Chiwanda Kaminga, Sixiang Cheng, Huilan Xu

**Affiliations:** 1 Department of Social Medicine and Health Management Xiangya School of Public Health Central South University Changsha China; 2 School of Acupuncture and Tuina Shandong University of Traditional Chinese Medicine Jinan China; 3 Department of Mathematics and Statistics Mzuzu University Luwinga Malawi; 4 Department of Epidemiology and Health Statistics Xiangya School of Public Health Central South University Changsha China

**Keywords:** COVID-19, health literacy, eHealth literacy, COVID-19–related health behavior questionnaire, Chinese college students

## Abstract

**Background:**

During the COVID-19 pandemic, the internet has significantly spread information, providing people with knowledge and advice about health protection regarding COVID-19. While a previous study demonstrated that health and eHealth literacy are related to COVID-19 prevention behaviors, few studies have focused on the relationship between health literacy, eHealth literacy, and COVID-19–related health behaviors. The latter includes not only preventative behaviors but also conventional health behaviors.

**Objective:**

The objective of this study was to develop and verify a COVID-19–related health behavior questionnaire, explore its status and structure, and examine the associations between these behaviors and participants’ health literacy and eHealth literacy.

**Methods:**

A snowball sampling method was adopted to recruit participants to complete anonymous cross-sectional questionnaire surveys online that assessed sociodemographic information, self-reported coronavirus knowledge, health literacy, eHealth literacy, and COVID-19–related health behaviors.

**Results:**

Of 1873 college students who were recruited, 781 (41.7%) had adequate health literacy; the mean eHealth literacy score was 30.16 (SD 6.31). The COVID-19–related health behavior questionnaire presented a two-factor structure—COVID-19–specific precautionary behaviors and conventional health behaviors—with satisfactory fit indices and internal consistency (Cronbach α=.79). The mean score of COVID-19–related health behaviors was 53.77 (SD 8.03), and scores differed significantly (*P*<.05) with respect to residence, college year, academic major, family economic level, self-reported health status, having a family member or friend infected with coronavirus, and health literacy level. Linear regression analysis showed that health literacy and eHealth literacy were positively associated with COVID-19–specific precautionary behaviors (*β*_health literacy_=.149, *β*_eHealth literacy_=.368; *P*<.001) and conventional health behaviors (*β*_health literacy_=.219, *β*_eHealth literacy_=.277; *P*<.001).

**Conclusions:**

The COVID-19–related health behavior questionnaire was a valid and reliable measure for assessing health behaviors during the pandemic. College students with higher health literacy and eHealth literacy can more actively adopt COVID-19–related health behaviors. Additionally, compared to health literacy, eHealth literacy is more closely related to COVID-19–related health behaviors. Public intervention measures based on health and eHealth literacy are required to promote COVID-19–related health behaviors during the pandemic, which may be helpful to reduce the risk of COVID-19 infection among college students.

## Introduction

### COVID-19–Related Health Behaviors

The COVID-19 pandemic, a major public health emergency, has become a serious burden not only for China but also worldwide in terms of threatening people’s lives as well as their mental health. At present, governments and institutions worldwide have introduced a series of policies to curb the spread of the epidemic, which have prevented and delayed the spread of COVID-19 to a certain extent [1]. However, vigilance continues to be required as global spread of COVID-19 is still very grim. World Health Organization data showed that as of March 22, 2021, the number of confirmed cases worldwide was 123,968,187, and 2,729,330 people have died worldwide [2]. Fortunately, new coronavirus vaccines have been developed. However, as current supply is insufficient, with potentially inconsistent quality among the different vaccines and manufacturers, many people have expressed suspicion and fear regarding vaccines [3] . Therefore, public preventive measures remain key to slowing the spread of COVID-19.

As a major public health event, the COVID-19 pandemic has required action from both governments and individuals. Since the COVID-19 outbreak, the Chinese government has implemented family-based isolation measures and other COVID-19 response measures, such as disease prevention strategies and advice for psychological adjustment [4]. In January 2020, the Guidelines for Public Protection Against Pneumonia Caused by the Novel Coronavirus Infection, which were compiled by the Chinese Center for Disease Control and Prevention (China CDC) [5], were provided to the public for free. These guidelines, which contain COVID-19 knowledge and prevention advice, such as personal protection, home medical observation, and psychological counseling, have been widely disseminated on online platforms such as social media. In addition to government measures, personal health behaviors also play a vital role in curbing the spread of COVID-19. Many previous studies have shown that public compliance with preventive behaviors can help reduce the spread of COVID-19 [6-8]. In addition to preventive behaviors, people’s conventional health behaviors, such as physical exercise and diet, also play an important role in maintaining physical and mental health during the COVID-19 epidemic. Therefore, it is important to understand the characteristics of COVID-19–related health behaviors. In this study, we define COVID-19–related health behaviors as those that are in response to the perceived threat of COVID-19, including government-recommended preventive behaviors and self-adaptive conventional health behaviors. It is noted that few studies have developed specific tools by which to assess the degree of public COVID-19–related health behavior participation. A previous study in the United States developed a 9-item scale based on health behavior recommendations proposed by the US Centers for Disease Control and Prevention to assess individual health behaviors [9]. However, due to the different national conditions of the United States and China, this tool may not be suitable for the Chinese population. Through a literature review, we found that most research on COVID-19 health behavior in China was conducted using a single item or a count of checklist items; the reliability and validity of such approaches remain unclear. Therefore, objectively measuring people’s COVID-19–related health behaviors and conducting research into the factors related to such behaviors may help formulate effective interventions for COVID-19 prevention and control.

### Health Literacy

Due to the implementation of family isolation, people have had to rely on internet searches to obtain relevant COVID-19 health information (eg, prevention information and control guidelines), which may be associated with their COVID-19–related health behaviors. However, early studies have shown that although the internet can provide resources for individuals seeking health information, people do not always correctly utilize these resources to solve health problems [10]. Additionally, previous studies have demonstrated that individuals with greater health literacy may more precisely distinguish the authenticity and accuracy of COVID-19–related information on media platforms and have superior understanding of health information in general [11,12].

Health literacy is defined as an individual’s ability to acquire, process, and understand basic health information and services in order to make appropriate health decisions [13]. Such literacy has long been associated with indicators of health [14]. A previous study showed that health literacy is associated with depression and health-related quality of life during the COVID-19 pandemic [15]. Specifically, medical students with higher health literacy have been shown to exhibit less fear of COVID-19 [16]. Additionally, adolescents’ health literacy was shown to be significantly related to handwashing-related knowledge and behavior during the pandemic’s early stages in Norway [17]. These examples indirectly illustrate that individuals with adequate health literacy are more likely to adopt COVID-19–related health behaviors, and the health literacy skill framework suggests that individuals’ health behaviors were affected by their health literacy skills [18]. Therefore, we hypothesized that health literacy would be positively correlated with COVID-19–related health behaviors.

### eHealth Literacy

In contrast, eHealth literacy specifically should be emphasized because while not everyone has a high level of health literacy during the COVID-19 crisis, they can still search, acquire, and utilize health information through the internet, allowing them to adopt relevant health behaviors based on this information. Health and eHealth literacy have distinct but related definitions, as both emphasize the individual’s ability to collect, evaluate, and utilize health information; however, unlike health literacy, eHealth literacy focuses on the ability to obtain and apply online health information via electronic media [10]. Therefore, the hypothesis concerning the relationship between health literacy and COVID-19–related health behaviors may be extended to include eHealth literacy, even though specific associations may be different for the two types of literacy.

Although previous studies emphasized the importance of eHealth literacy during COVID-19 [19,20]—and in the research of netizens [21], health care workers [22], and nursing students [23] it was found that eHealth literacy was related to COVID-19 prevention behavior—few studies have focused on the relationship between eHealth literacy and other COVID-19–related health behaviors other than preventive behaviors, especially among college students. Conventional health behaviors, such as physical exercise and nutritious eating, are also COVID-19–related health behaviors. In addition, a literature review indicated that eHealth literacy, healthy behaviors, and health outcomes are related [24]. Finally, although a previous study found that college students with a low level of eHealth literacy engaged in poor health-promoting behaviors [25], this relationship has not been adequately explored in a pandemic environment. Therefore, it is necessary to further analyze the relationship between eHealth literacy and COVID-19–related health behaviors. Overall, this online cross-sectional survey in China had two purposes: (1) develop and verify a COVID-19–related health behavior questionnaire and explore its status and structure and (2) study the relationship between health literacy, eHealth literacy, and COVID-19–related health behaviors.

## Methods

### Study Participants

A snowball sampling method was used to recruit the subjects. First, we published a recruitment announcement on QQ, which is a social media platform commonly used by Chinese college students. After screening, we identified 20 college students as the first group of participants from 20 universities across five regions of eastern, western, southern, northern, and central China. Each region included four universities to ensure that the sample broadly represented college students from all of China’s regions.

Then, these participants anonymously completed an internet-based, cross-sectional questionnaire and were asked to send the link to the blank questionnaire to their classmates. Participation was voluntary and no financial incentive was given for participating in the study. The purpose of both the survey and informed consent form was displayed on the first page of the online questionnaire. If they agreed to participate, subjects could click the *Next* button to complete the questionnaire; if they chose not to participate, they could click the *X* button. The questionnaire could be completed in 15 minutes, and there was only one chance for a given internet protocol address from which to complete the questionnaire.

This study was approved by the Ethics Committee of Xiangya School of Public Health of Central South University. The survey started on May 10, 2020, and ended on May 20, 2020, recruiting a total of 2152 participants. Of these, 279 were excluded for their illogical completion of the questionnaire (eg, they took less than 5 minutes to complete it or their age was under 10 years). Finally, 1873 valid questionnaires were considered for our data analysis.

### Measurements

#### Health Literacy

Health literacy was measured using the National Health Literacy Survey Questionnaire, as compiled by the China Health Education Center [26]. The overall Cronbach α of the scale was .95, and the Spearman-Brown coefficient was .94, indicating strong psychometric properties with minor measurement invariance [27]. The questionnaire was divided into three aspects: (1) basic health knowledge and literacy, (2) healthy lifestyle and behavior literacy, and (3) basic health skills literacy. These aspects covered the following six types of health problem literacy: scientific health, infectious disease prevention, chronic disease prevention, safety and emergency, basic medical information, and health-related information. The questionnaire consisted of 50 questions, including 34 multiple-choice questions and 16 multiple-answer questions (Multimedia Appendix 1). The total score ranged from 0 to 66 points, with 1 point per multiple-choice question and 2 points per multiple-answer question. A higher total score indicated a higher health literacy level; specifically, a total score of 53 or more indicated that the individual had adequate health literacy [28]. The Cronbach α coefficient for the questionnaire in this study was .83.

#### eHealth Literacy

eHealth literacy was measured using the Chinese version [29] of the eHealth Literacy Scale for College Students, originally developed by Norman and Skinner [30] to assess an individual’s abilities in terms of accessing, understanding, and evaluating health information from electronic media and utilizing this information to solve health problems. The scale has eight items in total; each item was rated on a scale of 1 (disagree) to 5 (agree) (Multimedia Appendix 1). The total score ranged from 8 to 40; the higher the score, the higher the eHealth literacy. The Cronbach α coefficient for the scale in this study was .92.

#### COVID-19–Related Health Behaviors

We designed a questionnaire for measuring COVID-19–related health behaviors. First, we conducted an extensive literature review and identified 2 categories—government-recommended preventive behaviors and self-adaptive conventional health behaviors—to serve as the questionnaire’s dimensions and overall framework. Based on the literature and the Guidelines for Public Protection Against Pneumonia Caused by the Novel Coronavirus Infection [5], we designed 24 items, 12 in each of the two categories. Second, an expert team composed of emergency management experts, public health experts, psychologists, respiratory doctors, and nurses evaluated the 24 items. According to their recommendations, seven items were deleted from the self-adaptive conventional health behaviors category, while three items were added to the preventive behaviors dimension because the experts believed that behaviors to prevent infection were the most important to adopt during the COVID-19 pandemic. Third, the 20 remaining items were sent to a different expert group, consistent with the previous group’s composition, for review. According to the group’s opinions, five items were deleted from the preventive behaviors category.

Finally, a 15-item questionnaire was formed (Multimedia Appendix 1). We recruited 85 college students (41 males [48%] and 44 females [52%] with mean age of 20.1 years, SD 1.4) to conduct an online preliminary assessment. A 5-point Likert scale was used to test whether individuals understood each item’s description, and they were asked whether they thought the item needed to be modified. The results showed that all respondents understood the descriptions (mean 4.48, SD 0.30), as all respondents indicated that no changes were required.

The questionnaire’s 15 items focused on problem solving, such as wearing masks and handwashing; seeking social support, such as contacting relatives and friends online; distraction, denial, or avoidance, such as smoking and drinking; and positive appraisals, such as physical exercise and maintaining a reasonable diet. Participants were asked to indicate their practice of each behavior over the past 2 weeks. Each item used a 5-level scoring method: 1 (none of the time), 2 (a small amount of the time), 3 (sometimes), 4 (most of the time), and 5 (almost all of the time). It should be noted that the scores for smoking and drinking were reversed. Therefore, for this questionnaire, the higher the score, the healthier and more positive a COVID-19–related health behavior was considered.

#### Covariates

Covariates included sociodemographic characteristics (ie, age, gender, residence, college year, academic major, and self-rated family economic level) and information related to COVID-19 (ie, self-reported health status, family members or friends infected with the coronavirus, and self-reported coronavirus knowledge level) (Multimedia Appendix 1). The self-reported COVID-19 knowledge level was evaluated via five questions, regarding the source of COVID-19 infection, incubation period, main transmission route, susceptible population, and primary clinical manifestations (Multimedia Appendix 1). The questions and answers were based on the COVID-19 Diagnosis and Treatment Protocol (Tentative Version Seven) that was issued by the National Health Commission of China [31]. For these questions, 1 point was assigned to correct answers and no points were assigned to answers that were either incorrect or unknown. The total score ranged from 0 to 5; the higher the score, the higher the coronavirus knowledge level.

### Statistical Analysis

Descriptive analysis was used to summarize participants’ sociodemographic information and other variables. Categorical variables were described using a frequency and percentage, whereas continuous variables were described by mean and SD. Then, to evaluate the factor structure and structural validity of the COVID-19–related health behavior questionnaire, a principal component exploratory factor analysis (EFA) with a varimax rotation and a confirmatory factor analysis (CFA) were used. It should be noted that we randomly divided the total samples into Sample A (511 men and 426 women), which was used for EFA, and Sample B (458 men and 478 women), which was used for CFA.

The Kaiser-Meyer-Olkin (KMO>0.50) measure of sampling adequacy and the Bartlett test of sphericity (*P* value of the Bartlett test <.05) were used to assess the questionnaire’s suitability for factor analysis [32]. Eigenvalues of greater than 1 were used to determine the number of factors, and a factor loading of greater than 0.50 was regarded as the criterion for keeping items.

Then, CFA, using SPSS Amos, version 23.0 (IBM Corp), was conducted to examine the factor structure of the COVID-19–related health behavior questionnaire. The CFA’s goodness of fit was evaluated using the following indicators: goodness-of-fit index (GFI >0.90), adjusted GFI (AGFI >0.90), root mean square error of approximation (RMSEA <0.80), comparative fit index (CFI >0.90), Tucker-Lewis index (TLI >0.90), and standardized root mean square residual (SRMR <0.80) [33]. The Cronbach α coefficient was used to evaluate the questionnaire’s internal consistency reliability.

The Student *t* test, or one-way analysis of variance (ANOVA), was used to evaluate the statistical differences in the distribution of COVID-19–related health behaviors across different sociodemographic characteristics and health literacy levels. Cohen *d* was used to evaluate effect size. Pearson correlations were used to quantify the bivariate associations between self-reported coronavirus knowledge level, health literacy, eHealth literacy, and COVID-19–related health behaviors. Hierarchical linear regression was used to analyze the relationship between health literacy, eHealth literacy, and COVID-19–related health behaviors. Correlation magnitudes were reported as standardized regression coefficients (*β*). SPSS, version 23.0 (IBM Corp), was used to perform statistical analyses. All statistical significance levels were set at α=.05, and the statistical tests were two-tailed.

## Results

### Participant Characteristics

The mean age of the participants was 19.6 years (SD 1.8), ranging from 18 to 25 years. Of the 1873 participants, 1505 (80.4%) had good self-reported health status, while 66 (3.5%) had family members or friends infected with coronavirus. The mean self-reported coronavirus knowledge level score was 4.11 (SD 1.18), and nearly half of the participants (903/1873, 48.2%) correctly answered five COVID-19 knowledge questions. The mean health literacy score was 50.05 (SD 9.55), and 781 out of 1873 participants (41.7%) had adequate health literacy. The mean eHealth literacy score was 30.16 (SD 6.31). For further details, see Table 1.

**Table 1:** Descriptive statistics of sociodemographic characteristics of the study participants.

Characteristic		Participants (N=1873), n (%)
**Gender**		
	Male	969 (51.7)
	Female	904 (48.3)
**Residence**		
	Urban	871 (46.5)
	Rural	1002 (53.5)
**College year**		
	Freshman	394 (21.0)
	Sophomore	594 (31.7)
	Junior	507 (27.1)
	Senior	378 (20.2)
**Academic major**		
	Medicine	851 (45.4)
	Others	1022 (54.6)
**Family economic level**		
	High	568 (30.3)
	Medium	1114 (59.5)
	Low	191 (10.2)
**Self-reported health status**		
	Good	1505 (80.4)
	Medium	336 (17.9)
	Bad	32 (1.7)
**Family member or friend infected with coronavirus**		
	Yes	66 (3.5)
	No	1807 (96.5)
**Self-reported coronavirus knowledge level**		
	0 correct responses	34 (1.8)
	1 correct response	69 (3.7)
	2 correct responses	96 (5.1)
	3 correct responses	156 (8.3)
	4 correct responses	615 (32.8)
	5 correct responses	903 (48.2)
**Health literacy level**		
	Inadequate	1092 (58.3)
	Adequate	781 (41.7)

### Internal Consistency and Structural Validity of the COVID-19–Related Health Behavior Questionnaire

First, we used the total sample for a presupposed two-factor structure analysis: 10 items for government-recommended preventive behaviors and five items for self-adaptive conventional health behaviors. However, the two-factor model for a 15-item questionnaire did not satisfactorily fit the data in the total sample (GFI=0.804; AGFI=0.735; RMSEA=0.131; CFI=0.658; TLI=0.597; SRMR=0.127). Therefore, we performed an EFA and a CFA. Table 2 shows the EFA results for each item of the COVID-19–related health behavior questionnaire. The value of the KMO test was 0.85, and the *P* value of Bartlett test was <.001. For each item, the factor loading ranged from 0.50 to 0.75 (all ≥0.50), such that the conditions for conducting EFA were satisfied. The results revealed two factors with eigenvalues greater than 1, accounting for 45.96% of the total variance. Factor 1 was comprised of eight items; its eigenvalue was 4.40 and it explained 29.34% of the variance. Factor 2 was comprised of seven items; its eigenvalue was 2.49 and it explained 16.62% of the variance.

Next, we performed CFA with Sample B. The results showed that a two-factor model had adequate goodness of fit (GFI=0.948; AGFI=0.919; RMSEA=0.068; CFI=0.924; TLI=0.909; SRMR=0.057). Based on the items that loaded on each factor, we preliminarily named the two factors COVID-19–specific precautionary behaviors, which contained eight items related to prevention, and conventional health behaviors, which contained seven items related to conventional health behaviors. Among the items, the highest score was for *cover your mouth and nose when you cough or sneeze* and the lowest score was for *insist on physical exercise*. For the 15 items in the total sample, the Cronbach α internal reliability coefficient was .79. 

**Table 2:** Exploratory factor analysis and descriptive statistics of the COVID-19–related health behavior questionnaire for college students.

Item	Sample A factor loading (n=937)		Total sample score^a^ (N=1873), mean (SD)
	Factor 1	Factor 2	
Maintain hand hygiene	0.75	0.20	3.84 (0.96)
Open windows for ventilation to maintain air circulation	0.70	0.16	3.87 (0.96)
Wear a mask when going out	0.69	0.06	4.10 (0.97)
Reduce instances of going to public places	0.63	0.30	3.70 (0.95)
Cover your mouth and nose when you cough or sneeze	0.61	0.19	4.28 (0.96)
Disinfect daily necessities	0.60	0.36	3.52 (1.03)
Contact relatives and friends online	0.52	0.30	3.50 (0.98)
Communicate or confide with others when you are in trouble	0.51	0.22	3.54 (1.07)
Maintain adequate nutrition and balanced diet	0.23	0.66	2.86 (1.06)
Drink alcohol because of COVID-19	0.26	0.65	4.11 (1.16)
Insist on physical exercise	0.08	0.64	2.72 (1.21)
Smoke because of COVID-19	0.35	0.63	4.18 (1.28)
Follow the latest developments on COVID-19	0.30	0.63	3.07 (1.07)
Take body temperature frequently	0.25	0.53	3.24 (1.11)
Guarantee good sleep	0.34	0.50	3.25 (1.09)

^a^Responses were based on a 5-level scoring method: 1 (none of the time), 2 (a small amount of the time), 3 (sometimes), 4 (most of the time), and 5 (almost all of the time).

### The Distribution of COVID-19–Related Health Behaviors Across Different Characteristics

The mean scores of COVID-19–related health behaviors, COVID-19–specific precautionary behaviors, and conventional health behaviors were 53.77 (SD 8.03), 30.36 (SD 5.17), and 23.41 (SD 3.87), respectively. The results of *t* tests and ANOVAs showed that the differences in COVID-19–related health behavior scores and COVID-19–specific precautionary behavior scores among college students with different genders, residences, college years, academic majors, family economic levels, self-reported health statuses, family members or friends infected with coronavirus, and health literacy levels were statistically significant (*P*<.05). In addition, there were also significant differences in conventional health behavior scores among the college students with different residences, college years, family economic levels, self-reported health statuses, family members or friends infected with coronavirus, and health literacy levels (*P*<.05). Further details are displayed in Multimedia Appendix 2.

### Associations Between Self-Reported Coronavirus Knowledge Level, Health Literacy, eHealth Literacy, and COVID-19–Related Health Behaviors

Table 3 shows that self-reported coronavirus knowledge levels were positively associated with health literacy (*r*=0.162; *P*<.001), eHealth literacy (*r*=0.220; *P*<.001), and COVID-19–related health behaviors (*r*=0.244; *P*<.001). Additionally, health literacy was positively associated with eHealth literacy (*r*=0.270; *P*<.001) and COVID-19–related health behaviors (*r*=0.338; *P*<.001), while eHealth literacy was positively associated with COVID-19–related health behaviors (*r*=0.476; *P*<.001).

**Table 3:** Correlation matrix (Pearson *r* and two-tailed *P* value^a^) of self-reported coronavirus knowledge level, health literacy, eHealth literacy, and health and precautionary behaviors.

Variable		Self-reported coronavirus knowledge level	Health literacy		eHealth literacy		COVID-19–related health behaviors		COVID-19–specific precautionary behaviors		Conventional health behaviors	
**Self-reported coronavirus knowledge level**												
	*r*	1		0.162		0.220		0.244		0.283	0.129	
	*P* value	—^b^		<.001		<.001		<.001		<.001	<.001	
**Health literacy**												
	*r*	0.162		1		0.270		0.338		0.288	0.316	
	*P* value	<.001		—		<.001		<.001		<.001	<.001	
**eHealth literacy**												
	*r*	0.220		0.270		1		0.476		0.471	0.357	
	*P* value	<.001		<.001		—		<.001		<.001	<.001	
**COVID-19–related health behaviors**												
	*r*	0.244		0.338		0.476		1		0.918	0.848	
	*P* value	<.001		<.001		<.001		—		<.001	<.001	
**COVID-19–specific precautionary behaviors**												
	*r*	0.283		0.288		0.471		0.918		1	0.568	
	*P* value	<.001		<.001		<.001		<.001		—	<.001	
**Conventional health behaviors**												
	*r*	0.129		0.316		0.357		0.848		0.568	1	
	*P* value	<.001		<.001		<.001		<.001		<.001	—	

^a^For all associations, the correlation is significant at a significance level of *.*05 (two-tailed).

^b^Not applicable.

### Factors Associated With COVID-19–Related Health Behaviors: Multivariable Analyses

Hierarchical linear regression analysis was conducted with the score of the COVID-19–related health behaviors (COVID-19–specific precautionary behaviors and conventional health behaviors) as the dependent variable, while the independent variables consisted of sociodemographic characteristics, health literacy, and eHealth literacy. In Block 1, the following sociodemographic characteristics were entered first, accounting for 17.4% of the variance in COVID-19–specific precautionary behaviors (*R*^2^=0.174; *F*_13, 1859_=33.890; *P*<.001) and 7.4% of the variance in conventional health behaviors (*R*^2^=0.074; *F*_13, 1859_=13.393; *P*<.001): gender, residence, college year, academic major, family economic level, self-reported health status, having a family member or friend infected with coronavirus, and self-reported coronavirus knowledge level. The score for health literacy was entered into Block 2 and contributed to explaining an additional 4.2% of the variance in COVID-19–specific precautionary behaviors (*R*^2^=0.216; *F*_14, 1858_=40.692; *P*<.001) and an additional 6.4% of the variance in conventional health behaviors (*R*^2^=0.138; *F*_14, 1858_=24.045; *P*<.001). Finally, the score for eHealth literacy was entered into Block 3 and contributed to explaining an additional 11.5% of the variance in COVID-19–specific precautionary behaviors (*R*^2^=0.332; *F*_15, 1857_=67.449; *P*<.001) and an additional 6.5% of the variance in conventional health behaviors (*R*^2^=0.203; *F*_14, 1858_=35.083; *P*<.001). The linear regression results are presented in Multimedia Appendix 3.

Multimedia Appendix 3 illustrates that the listed sociodemographic characteristics, other than residence and academic major, were related to COVID-19–related health behaviors. Overall, participants were more likely to have superior COVID-19–specific precautionary behavior scores if they were female versus male (*β*=.153; *P*<.001), freshmen versus seniors (*β*=.094; *P*<.001), juniors versus seniors (*β*=–.061; *P*=.02), from a family with a medium versus low economic level (*β*=.122; *P*<.001), had a self-reported health status that was good versus bad (*β*=.205; *P*=.001), had a family member or friend infected with coronavirus versus not infected (*β*=.095; *P*<.001), and had a higher self-reported coronavirus knowledge level (*β*=.156; *P*<.001). Conventional health behaviors were more likely to be performed by individuals who were sophomores versus seniors (*β*=.074; *P*<.001), from a family with a high or medium economic level (high vs low: *β*=.082, *P*=.03; medium vs low: *β*=.077, *P*=.03), had good or medium self-reported health status (good vs bad: *β*=.273, *P*<.001; medium vs bad: *β*=.171, *P*=.008).

After adjusting for covariates, both health literacy and eHealth literacy were positively associated with COVID-19–specific precautionary behaviors (*β*_health literacy_=.149, *β*_eHealth literacy_=.368; *P*<.001) and conventional health behaviors (*β*_health literacy_=.219, *β*_eHealth literacy_=.277; *P*<.001). Among the related factors, eHealth literacy was most strongly associated with COVID-19–related health behaviors.

## Discussion

### Principal Findings

Based on the China CDC’s guidelines and expert advice, we developed and verified a COVID-19–related health behavior questionnaire among college students. The questionnaire was found to be valid and reliable for assessing health behaviors during the pandemic. This study also examined whether health literacy and eHealth literacy were associated with the COVID-19–related health behaviors of college students. To the best of our knowledge, this is one of few studies to consider the relationship between these factors for this population group. The study results showed that health and eHealth literacy were positively related to COVID-19–related health behaviors among college students.

The factor analysis showed that the COVID-19–related health behavior questionnaire had a two-factor structure, which is consistent with the two-factor structure we had previously assumed. That is, the eight items for Factor 1 primarily reflected individual protective measures, such as wearing a mask, maintaining hand hygiene, and reducing the amount of time that one leaves the house. Therefore, Factor 1 was named *COVID-19–specific precautionary behaviors*. Furthermore, the seven items for Factor 2 reflected individuals’ conventional health behaviors, such as physical exercise and diet during the COVID-19 pandemic; therefore, it was named *conventional health behaviors*. In addition, the results showed that the internal consistency reliability of the questionnaire was adequate. Based on these two relatively simple psychometric indicators, the COVID-19–related health behavior questionnaire appears to have satisfactory internal consistency reliability and construct validity in this context, so that it could be used among Chinese college students. This questionnaire may be useful for government officials and school health educators to assess the COVID-19–related health behaviors of college students in order to carry out targeted interventions.

The results of the descriptive analysis showed that college students’ COVID-19–specific precautionary behaviors were high, and the scores of eight items were all higher than 3.5, among which the score of *cover your mouth and nose when you cough or sneeze* was the highest. A European study of adolescents obtained similar results, namely that adolescents seemed to be generally aware of the recommendations regarding protective behaviors during the COVID-19 crisis [17]. In contrast, this study found that college students’ conventional health behaviors were poor, especially in terms of their scores for physical exercise and maintaining both adequate nutrition and a balanced diet, which were lower than the median (3 points). Zhao et al also found that after the COVID-19 outbreak, only a small number of Chinese people participated in physical exercise, and most consumed vegetables and fruits even less frequently than usual each week [34]. This may be related to affective disorders, such as anxiety and depression during the COVID-19 pandemic. A cross-sectional study suggested that less physical exercise was found to negatively impact anxiety and depressive symptoms in college students during the COVID-19 pandemic [35]. However, in turn, anxiety and depression can cause a lack of motivation to exercise and eat healthily. Thus, this study indirectly reflects the changes that the COVID-19 pandemic caused in people’s lifestyles and behaviors. Additionally, previous studies showed that unhealthy behaviors were related to COVID-19 mortality rates and hospital admissions, and although they provided some specific lifestyle recommendations [36,37], few studies have analyzed intervenable factors related to COVID-19–related health behaviors during the pandemic.

In this study, after adjusting for sociodemographic characteristics, health and eHealth literacy were shown to be significantly related to COVID-19–related health behaviors, which supports the findings of previous research on the relationship between health literacy and COVID-19–related attitudes and behaviors [38]. For example, a study in Hong Kong demonstrated that health literacy significantly and positively correlated with hand hygiene habits in older persons [39], consistent with a study of adolescents [17], indicating that health literacy is closely related to COVID-19 health protection behaviors. In addition, health literacy’s positive relationship with physical exercise, diet, and sleep has been confirmed by numerous studies [40-42]; likewise, this study confirmed this relationship. This indicates that, even during the COVID-19 pandemic, health literacy remains related to healthy behaviors. Nevertheless, these results cannot explain the causal relationship between health literacy and COVID-19–related health behaviors. However, based on previous research experience [43], individuals with a high level of health literacy are more likely to adopt positive behaviors in their response to the COVID-19 pandemic. Additionally, although it was obviously difficult to improve the health literacy of individuals on such a short-term basis, significantly curbing the disease’s spread will involve finding new intervention strategies and incorporating the abundance of information related to COVID-19 on the internet.

This study showed that eHealth literacy was also positively correlated with college students’ COVID-19–related health behaviors. Similarly, a previous study illustrated that people who had obtained more health information online related to COVID-19 were more frequently involved in various types of preventive behaviors [44]. Researchers have attributed this association to risk perception; COVID-19 pandemic information on the internet exacerbates concerns and may prompt users to adopt protective behaviors [45]. Further, a related study of college students reported that their eHealth literacy is associated with regular exercise, healthy eating, and routine sleep [46]. Moreover, a study found that eHealth literacy is related to disease prevention behaviors, such as finding vaccination information [47]. These results indirectly support the association between eHealth literacy and COVID-19–related health behaviors. Furthermore, a recent study showed that eHealth literacy is related to preventive behaviors in relation to the COVID-19 pandemic [21], consistent with findings in this study. However, the previous study had notably few people under the age of 20 years, so that this study, which included a substantial number of subjects under this age, further expanded upon their findings.

This study also found that a higher level of eHealth literacy was associated with superior conventional health behaviors, indicating that college students with a higher level of eHealth literacy in China could maintain healthy lifestyles during the COVID-19 pandemic. While previous studies have paid more attention to health protection behaviors, few have examined other behaviors during the COVID-19 pandemic, such as exercise, diet, sleep, smoking, and consuming alcohol. For example, while the significance of COVID-19–specific precautionary behaviors focuses on reducing the possibility of an individual becoming infected with the coronavirus, conventional health behaviors provide for greater reflection in an individual’s daily life during this period of isolation from family due to COVID-19 prevention and control measures. Conventional health behaviors emphasize the ability to adapt to life events, which is particularly important when promoting physical and mental health, such as maintaining resilience, reducing and avoiding the occurrence of other diseases, and relieving both mental pressures and stress. Therefore, this study suggests that improving health and eHealth literacy may help college students adopt healthy lifestyles and adapt to the isolation that has been caused by the COVID-19 pandemic.

Compared to health literacy, eHealth literacy was a stronger predictor of COVID-19–related health behaviors, which may be due to COVID-19 being a sudden infectious disease. That is, the nature of this disease causes people to lack sufficient understanding about it, which leads to a lack of knowledge about how to take preventive measures. Since the Chinese government implemented family isolation measures during the COVID-19 crisis, they published knowledge and guidelines on the internet about various COVID-19 prevention and control measures. A previous study showed that individuals with higher levels of eHealth literacy more often search for health information on the internet [48], which means that college students with a high level of eHealth literacy may acquire more COVID-19 information online, enabling them to adopt health behaviors. Considering that health literacy is the result of personal, long-term learning and behavioral practice [49], it is difficult to change this feature in a short-term period. Therefore, this study suggests that it is particularly important to pay more attention to improving individual eHealth literacy.

This study’s results have important value in terms of public health, especially concerning the prevention and control of COVID-19 in universities. Many universities around the world have resumed studies on campus, causing numerous students to gather on small campuses, which places greater pressure on the need for COVID-19 prevention and control. For instance, once an asymptomatic patient who is infected with the coronavirus appears at school, they could transmit the infection to other students, especially persons not practicing health protection measures. Since college students are the primary users of electronic media and the internet in general, the feasibility and practical effects of related prevention and control interventions through eHealth literacy are potentially significant.

Regarding limitations, snowball sampling is nonprobability sampling that is generally used when it is difficult to identify the members of the population. Since the Chinese government adopted the family segregation policy when we conducted the survey and schools did not open, it was difficult for us to use probability sampling when conducting the survey. Snowball sampling has the possibility of sampling and selection bias, which limits the extrapolation of our research. Second, a cross-sectional survey cannot explain the causal relationships among health literacy, eHealth literacy, and COVID-19–related health behaviors. Third, in the survey, we did not use a standardized questionnaire to assess participants’ knowledge of COVID-19, which may not adequately reflect knowledge of COVID-19. Recent research developed a health literacy scale related to the coronavirus [50]; future research could utilize this scale to obtain more in-depth understanding. Finally, the COVID-19–related health behavior questionnaire was compiled according to China’s cultural background as well as the state’s COVID-19 prevention and control guidelines. Thus, it may not be applicable to other countries, which limits the inferences one may make from these results.

### Conclusions

The COVID-19–related health behavior questionnaire was a valid and reliable measure for assessing health behaviors during the pandemic. The results of this study suggest that college students with higher health literacy and eHealth literacy can more actively adopt COVID-19–related health behaviors. Additionally, compared to health literacy, eHealth literacy is more closely related to COVID-19–related health behaviors. These findings are significant for COVID-19 prevention and control in the college environment, including for relevant health education activities or other intervention measures based on health and eHealth literacy that can be carried out in a timely manner to enhance COVID-19–related health behaviors and reduce the risk of infection among college students.

## Appendix

Multimedia Appendix 1 Study questionnaires.

Multimedia Appendix 2 Comparison of COVID-19–related health behaviors among college students with different demographic characteristics.

Multimedia Appendix 3 Factors associated with COVID-19–related health behaviors in Chinese college students.

## Abbreviations

AGFI: adjusted goodness-of-fit index
ANOVA: analysis of variance
CFA: confirmatory factor analysis
CFI: comparative fit index
China CDC: Chinese Center for Disease Control and Prevention
EFA: exploratory factor analysis
GFI: goodness-of-fit index
KMO: Kaiser-Meyer-Olkin
RMSEA: root mean square error of approximation
SRMR: standardized root mean square residual
TLI: Tucker-Lewis index

## References

[1] Hsiang S, Allen D, Annan-Phan S, Bell K, Bolliger I, Chong T, Druckenmiller H, Huang LY, Hultgren A, Krasovich E, Lau P, Lee J, Rolf E, Tseng J, Wu T (2020). The effect of large-scale anti-contagion policies on the COVID-19 pandemic. Nature.

[2] Coronavirus disease (COVID-19) weekly epidemiological update and weekly operational update. World Health Organization.

[3] Chou WS, Budenz A (2020). Considering emotion in COVID-19 vaccine communication: Addressing vaccine hesitancy and fostering vaccine confidence. Health Commun.

[4] Pan A, Liu L, Wang C, Guo H, Hao X, Wang Q, Huang J, He N, Yu H, Lin X, Wei S, Wu T (2020). Association of public health interventions with the epidemiology of the COVID-19 outbreak in Wuhan, China. JAMA.

[5] Chinese Center for Disease Control and Prevention (2020). Guidelines for Public Protection Against Pneumonia Caused by New Coronavirus Infection.

[6] Nguyen NPT, Hoang TD, Tran VT, Vu CT, Siewe Fodjo JN, Colebunders R, Dunne MP, Vo TV (2020). Preventive behavior of Vietnamese people in response to the COVID-19 pandemic. PLoS One.

[7] Chen X, Chen H (2020). Differences in preventive behaviors of COVID-19 between urban and rural residents: Lessons learned from a cross-sectional study in China. Int J Environ Res Public Health.

[8] Duan T, Jiang H, Deng X, Zhang Q, Wang F (2020). Government intervention, risk perception, and the adoption of protective action recommendations: Evidence from the COVID-19 prevention and control experience of China. Int J Environ Res Public Health.

[9] Toussaint LL, Cheadle AD, Fox J, Williams DR (2020). Clean and contain: Initial development of a measure of infection prevention behaviors during the COVID-19 pandemic. Ann Behav Med.

[10] Norman CD, Skinner HA (2006). eHealth literacy: Essential skills for consumer health in a networked world. J Med Internet Res.

[11] Zarocostas J (2020). How to fight an infodemic. Lancet.

[12] Baines D, Elliott R (2020). Defining misinformation, disinformation and malinformation: An urgent need for clarity during the COVID-19 infodemic. Discussion Papers 20-06. IDEAS.

[13] Berkman ND, Davis TC, McCormack L (2010). Health literacy: What is it?. J Health Commun.

[14] Greenhalgh T (2015). Health literacy: Towards system level solutions. BMJ.

[15] Nguyen HC, Nguyen MH, Do BN, Tran CQ, Nguyen TTP, Pham KM, Pham LV, Tran KV, Duong TT, Tran TV, Duong TH, Nguyen TT, Nguyen QH, Hoang TM, Nguyen KT, Pham TTM, Yang S, Chao JC, Duong TV (2020). People with suspected COVID-19 symptoms were more likely depressed and had lower health-related quality of life: The potential benefit of health literacy. J Clin Med.

[16] Nguyen HT, Do BN, Pham KM, Kim GB, Dam HTB, Nguyen TT, Nguyen TTP, Nguyen YH, Sørensen K, Pleasant A, Duong TV (2020). Fear of COVID-19 Scale-Associations of its scores with health literacy and health-related behaviors among medical students. Int J Environ Res Public Health.

[17] Riiser K, Helseth S, Haraldstad K, Torbjørnsen A, Richardsen KR (2020). Adolescents' health literacy, health protective measures, and health-related quality of life during the Covid-19 pandemic. PLoS One.

[18] Squiers L, Peinado S, Berkman N, Boudewyns V, McCormack L (2012). The health literacy skills framework. J Health Commun.

[19] Chong YY, Cheng HY, Chan HYL, Chien WT, Wong SYS (2020). COVID-19 pandemic, infodemic and the role of eHealth literacy. Int J Nurs Stud.

[20] Brørs G, Norman CD, Norekvål TM (2020). Accelerated importance of eHealth literacy in the COVID-19 outbreak and beyond. Eur J Cardiovasc Nurs.

[21] Li X, Liu Q (2020). Social media use, eHealth literacy, disease knowledge, and preventive behaviors in the COVID-19 pandemic: Cross-sectional study on Chinese netizens. J Med Internet Res.

[22] Do BN, Tran TV, Phan DT, Nguyen HC, Nguyen TTP, Nguyen HC, Ha TH, Dao HK, Trinh MV, Do TV, Nguyen HQ, Vo TT, Nguyen NPT, Tran CQ, Tran KV, Duong TT, Pham HX, Nguyen LV, Nguyen KT, Chang PWS, Duong TV (2020). Health literacy, eHealth literacy, adherence to infection prevention and control procedures, lifestyle changes, and suspected COVID-19 symptoms among health care workers during lockdown: Online survey. J Med Internet Res.

[23] Yuan T, Liu H, Li XD, Liu HR (2020). Factors affecting infection control behaviors to prevent COVID-19: An online survey of nursing students in Anhui, China in March and April 2020. Med Sci Monit.

[24] Neter E, Brainin E (2019). Association between health literacy, eHealth literacy, and health outcomes among patients with long-term conditions. Eur Psychol.

[25] Yang S, Luo Y, Chiang C (2017). The associations among individual factors, eHealth literacy, and health-promoting lifestyles among college students. J Med Internet Res.

[26] Li Y (2014). Introduction of 2012 Chinese residents health literacy monitoring program. Chin J Health Educ.

[27] Shen M, Hu M, Liu S, Chang Y, Sun Z (2015). Assessment of the Chinese Resident Health Literacy Scale in a population-based sample in South China. BMC Public Health.

[28] Wang W, Zhang Y, Lin B, Mei Y, Ping Z, Zhang Z (2020). The urban-rural disparity in the status and risk factors of health literacy: A cross-sectional survey in central China. Int J Environ Res Public Health.

[29] Guo S, Yu X, Sun Y, Nie D, Li X, Wang L (2013). Adaptation and evaluation of Chinese version of eHEALS and its usage among senior high school students. Chin J Health Educ.

[30] Norman CD, Skinner HA (2006). eHEALS: The eHealth Literacy Scale. J Med Internet Res.

[31] COVID-19 diagnosis and treatment protocol (tentative version 7). National Health Commission of the People's Republic of China.

[32] Williams B, Onsman A, Brown T (2010). Exploratory factor analysis: A five-step guide for novices. Australas J Paramedicine.

[33] Carrión G, Nitzl C, Roldán J, Latan H, Noonan R (2017). Mediation analyses in partial least squares structural equation modeling: Guidelines and empirical examples. Partial Least Squares Path Modeling: Basic Concepts, Methodological Issues and Applications.

[34] Hu Z, Lin X, Chiwanda Kaminga A, Xu H (2020). Impact of the COVID-19 epidemic on lifestyle behaviors and their association with subjective well-being among the general population in mainland China: Cross-sectional study. J Med Internet Res.

[35] Li Y, Zhao J, Ma Z, McReynolds LS, Lin D, Chen Z, Wang T, Wang D, Zhang Y, Zhang J, Fan F, Liu X (2021). Mental health among college students during the COVID-19 pandemic in China: A 2-wave longitudinal survey. J Affect Disord.

[36] Hamer M, Kivimäki M, Gale CR, Batty GD (2020). Lifestyle risk factors, inflammatory mechanisms, and COVID-19 hospitalization: A community-based cohort study of 387,109 adults in UK. Brain Behav Immun.

[37] Smirmaul BPC, Chamon RF, de Moraes FM, Rozin G, Moreira ASB, de Almeida R, Guimarães ST (2021). Lifestyle medicine during (and after) the COVID-19 pandemic. Am J Lifestyle Med.

[38] Wolf MS, Serper M, Opsasnick L, O'Conor RM, Curtis LM, Benavente JY, Wismer G, Batio S, Eifler M, Zheng P, Russell A, Arvanitis M, Ladner D, Kwasny M, Persell SD, Rowe T, Linder JA, Bailey SC (2020). Awareness, attitudes, and actions related to COVID-19 among adults with chronic conditions at the onset of the US outbreak: A cross-sectional survey. Ann Intern Med.

[39] Or PP, Wong BY, Chung JW (2020). To investigate the association between the health literacy and hand hygiene practices of the older adults to help them fight against infectious diseases in Hong Kong. Am J Infect Control.

[40] Klinker CD, Aaby A, Ringgaard LW, Hjort AV, Hawkins M, Maindal HT (2020). Health literacy is associated with health behaviors in students from vocational education and training schools: A Danish population-based survey. Int J Environ Res Public Health.

[41] Barsell DJ, Everhart RS, Miadich SA, Trujillo MA (2018). Examining health behaviors, health literacy, and self-efficacy in college students with chronic conditions. Am J Health Educ.

[42] Luo H, Chen Z, Bell R, Rafferty AP, Gaskins Little NR, Winterbauer N (2020). Health literacy and health behaviors among adults with prediabetes, 2016 Behavioral Risk Factor Surveillance System. Public Health Rep.

[43] Zhang L, Seale H, Wu S, Yang P, Zheng Y, Ma C, MacIntyre R, Wang Q (2014). Post-pandemic assessment of public knowledge, behavior, and skill on influenza prevention among the general population of Beijing, China. Int J Infect Dis.

[44] Li S, Feng B, Liao W, Pan W (2020). Internet use, risk awareness, and demographic characteristics associated with engagement in preventive behaviors and testing: Cross-sectional survey on COVID-19 in the United States. J Med Internet Res.

[45] Sun Z, Yang B, Zhang R, Cheng X (2020). Influencing factors of understanding COVID-19 risks and coping behaviors among the elderly population. Int J Environ Res Public Health.

[46] Tsukahara S, Yamaguchi S, Igarashi F, Uruma R, Ikuina N, Iwakura K, Koizumi K, Sato Y (2020). Association of eHealth literacy with lifestyle behaviors in university students: Questionnaire-based cross-sectional study. J Med Internet Res.

[47] Britt RK, Collins WB, Wilson K, Linnemeier G, Englebert AM (2017). eHealth literacy and health behaviors affecting modern college students: A pilot study of issues identified by the American College Health Association. J Med Internet Res.

[48] Schulz PJ, Fitzpatrick MA, Hess A, Sudbury-Riley L, Hartung U (2017). Effects of eHealth literacy on general practitioner consultations: A mediation analysis. J Med Internet Res.

[49] Edwards M, Wood F, Davies M, Edwards A (2012). The development of health literacy in patients with a long-term health condition: The health literacy pathway model. BMC Public Health.

[50] Okan O, Bollweg TM, Berens E, Hurrelmann K, Bauer U, Schaeffer D (2020). Coronavirus-related health literacy: A cross-sectional study in adults during the COVID-19 infodemic in Germany. Int J Environ Res Public Health.

