# Supplementary material for: Associations Between Health Literacy, eHealth Literacy, and COVID-19–Related Health Behaviors Among Chinese College Students: Cross-sectional Online Study
Source: J Med Internet Res. 2021 May 6;23(5):e25600. doi: 10.2196/25600 (PMC8104003; doi:10.2196/25600)
Supplement: Multimedia Appendix 1 [file jmir_v23i5e25600_app1.pdf]

Supplementary file 1. Survey questionnaire

Part 1：Health literacy

A True or False questions **(please choose "①" if you think the statement is correct, otherwise choose "②".)**

A01. The best way to prevent flu is to take antibiotics (anti-inflammatory drugs).

① Right ② Wrong

A02. Health foods are not medicines, nor can they replace medicines.

① Right ② Wrong

A03. Infusion is effective and fast, so you should first choose infusion after illness.

① Right ② Wrong

A04. Fruits and vegetables have similar nutrients, so you can replace eating vegetables into eating fruits.

① Right ② Wrong

A05. Normal people's body temperature can fluctuate up and down in one day, but the fluctuation range generally does not exceed 1 °C.

① Right ② Wrong

A06. Children and adolescents may also experience depression.

① Right ② Wrong

A07. "Long illness makes the patient a good doctor", patients with chronic diseases can adjust the treatment plan according to their own feelings.

① Right ② Wrong

A08. For health problems and diseases found during a health checkup, if there are no symptoms, no measures need to be taken immediately.

① Right ② Wrong

B Single choice questions **(Each question has only one correct choice. If you don't know, please select "④".)**

B01. Concerning the concept of health, the complete statement is:

① Health means being strong and disease-free.

② Health means good psychological quality and strong physique.

③ Health is not only the absence of disease, but also the sound state of physical, psychological and social adaptation.

④ I don't know.

B02. Which of the following ways can hepatitis B be transmitted to others?

① Work, eat, and swim with the patient or infected person

② Sexual behavior, blood transfusion, mother-to-child transmission.

③ Talk, shake hands, and hug with the patient or infected person.

④ I don't know.

B03. Concerning self-blood pressure measurement, which statement is wrong?
① Self-measured blood pressure has reference value for the diagnosis of hypertension.
② Regular self-monitoring of blood pressure in patients with hypertension can provide a basis for doctors to formulate treatment plans and evaluate the therapeutic effect.
③ As long as the blood pressure of hypertension patients is stable, they don't have to go to the clinic regularly for follow-up treatment.
④ I don't know.

B04. Concerning the danger of smoking, which statement is wrong?
① Tobacco dependence is a chronic addictive disease.
② Smoking can cause many kinds of chronic diseases.
③ Low tar cigarettes are less harmful than ordinary cigarettes.
④ I don't know.

B05. Which statements is not an early warning sign of cancer?
① Abnormal lump in the body.
② Unclear hematochezia.
③ Gain weight.
④ I don't know.

B06. After the occurrence of gas poisoning, how should the rescuers firstly deal with the people with gas poisoning?
① Give the patient water to drink.
② Move the patient to a ventilated place.
③ Call 120 for hospital treatment.
④ I don't know.

B07. Concerning the treatment of tuberculosis patients, which statement is correct?
① No preferential policies.
② Free anti-tuberculosis drugs provided by the state.
③ Free hospitalization.
④ I don't know.

B08. When engaging in toxic and hazardous operations, the staffs should:
① Wear overalls.
② Wear safety helmet.

③ Use personal occupational disease protection equipment.

④ I don't know.

B09. The main hazard of iodine deficiency is:

① Suffering from SARS.

② Influencing the developments of intelligence and growth.

③ Causing hypertension.

④ I don’t know.

B10. During vigorous activities, water will be lost due to sweating. In this case, it's better to drink:

① Boiled water.

② Sugary drinks.

③ Dilute brine.

④ I don’t know.

B11. Concerning the National basic public health service, which statement is wrong?

① Mainly conducted in large hospitals.

② Conducted in primary medical and health institutions.

③ People can enjoy it free of charge.

④ I don’t know.

B12. In which of the following cases, vaccination of children should be suspended?

① Crying.

② Have a cold/fever.

③ Within half an hour after meals.

④ I don’t know.

B13. When you have fever symptoms, the right thing to do is:

① Seeking a doctor in time

② According to past experiences, take antipyretics by yourself.

③ Observing the situation for a while.

④ I don’t know.

B14. When patients suffer from adverse reactions after taking medicine according to doctors’ treatment plans, the right thing to do is?

① You stop taking the medicine.

② Seeking a doctor.

③ Taking medicines continually.

④ I don’t know.

B15. If a strong infectious disease occurs in a certain place, the right thing to do is?

① This disease has nothing to do with me, and I don’t care about it.

② I would be concerned about the epidemic if I were a local.

③ Whether local or not, we need to pay attention to the change of epidemic situation.

④ I don’t know.

B16. Warning 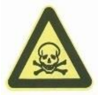 map means:

① The place is prone to fire.

② Explosives are present in an area of the site and are not allowed to approach.

③ This substance is toxic or there are toxic substances in the place.

④ I don’t know.

B17. The phone number of the national free health hotline is:

① 12302

② 120

③ 12320

④ I don’t know.

B18. Concerning seeking medical treatment, which statement is wrong?

① Tell the doctor as detailed as possible about the illness.

② If there are previous medical records, examination results, etc., it is best to carry them with you.

③ In order to make the doctor pay attention to it, we can describe the condition more seriously than it actually is.

④ I don’t know.

B19. If “OTC” is printed on a drug label, the drug shall be:

① A prescription drug，which must be prescribed by a doctor before it can be purchased.

② A Over-the-counter drug, which can be purchased without a doctor's prescription.

③ Health products.

④ I don’t know.

B20. Concerning window ventilation, which statement is wrong?

① Avoid colds by opening fewer or no windows in winter.

② Window ventilation can dilute bacteria and viruses in indoor air.

③ Opening windows and ventilation can let sunlight into the room, killing a variety of bacteria and viruses.

④ I don’t know.

B21. When measuring body temperature with a vitreous thermometer, the correct reading method is:

① Hold the mercury end of the thermometer and read it horizontally.

② Hold the glass end of the thermometer and read it vertically.

③ Hold the glass end of the thermometer and read it horizontally.

④ I don’t know.

B22. When blisters appear caused by mild scalds of the skin, which statement is right?

① Break the blisters, so that the recovery will be quick.

② Bubbles don’t need to be pricked if they’re small, but must be pricked if they’re large.

③ Do not prick blisters to avoid infection.

④ I don’t know.

B23. In case of fire, the correct escape method is:

① Hold your head in your hands, or wrap your head in your clothes, and rush out of the fire.

② Sprinkle water on your head and body, or wrap your body in a wet blanket, and rush out of the fire.

③ Beating at the flames with clothes, while evacuating from the fire.

④ I don’t know.

C Multiple choice questions **(Each question has two or more correct choices. If you don't know, please select ⑤.)**

C01. Which of the following are true about the ways to promote mental health?

① Keep an optimistic attitude towards life

② Set target within the scope of your own ability

③ Establish good interpersonal relations, and actively participate in social activities

④ Solve the worry through smoking, drinking

⑤ I don't know

C02. Which of the following are true about seeking medical advice?

① Not all diseases can be cured

② As long as going to hospital, the health status will be improved

③ A hospital is the place of healing, it is the hospital's responsibility not to cure the disease

④ Sickness and death are natural, rationally treated the diagnosis and treatment results

⑤ I don't know

C03. Which of the following are true about liver?

① Secrete bile

② Detoxification function

③ Most important digestive organ

④ The liver has one on each side

⑤ I don't know

C04. What should parents do while children appear fever, rash and other symptoms?

① Go to the hospital in time

② Stop going to kindergarten

③ Inform the teacher of the kindergarten in time

④ Let the child go to kindergarten as usual

⑤ I don't know

C05. What information should be noted on the package while buying packaged food?

① Production date

② Expiration date

③ Nutrition facts

④ Manufacturer

⑤ I don't know

C06. What should be done if dead or sick animals are found?

① no killing, no processing

② Not for sale, not for transport

③ don't eat

④ eating after cooking thoroughly

⑤ I don't know

C07. What measures should be taken for patients suffering from respiratory or cardiac arrest?

① Artificial respiration

② Chest compressions

③ Call the emergency number

④ Give hypertension treatment drugs

⑤ I don't know

C08. The benefits of eating tofu, soy milk and other soy products:

① Good for health

② Good for patients with cardiovascular diseases

③ Increase protein intake

④ Prevent excessive consumption of meat

⑤ I don't know

C09. The health benefits of exercise:

① Maintain a reasonable weight

② Prevent chronic diseases

③ Reduce psychological pressure

④ Improve sleep quality

⑤ I don't know

C10. A newspaper said that any diabetic can be completely cured by taking a certain hypoglycemic product. Which of the following statements are true after seeing this information?

① The news is not believable

② This news is good, hurry to tell diabetes friends

③ Consult and verify it with the community doctor

④ Hurry to buy

⑤ I don't know

C11. When coughing or sneezing, the correct ways are:

① Use hands to cover your nose and mouth directly

② Use a handkerchief or tissue to cover mouth and nose

③ Cover nose and mouth with elbow

④ Don't cover mouth and nose

⑤ I don't know

C12. Which of the following statement about hospitalization are true?

① The longer length of hospitalization, the better effect of the treatment

② Treatment effect and length of hospitalization is not necessarily related

③ The length of hospitalization depends on the patient's condition

④ The short length of hospitalization is a sign of doctors' irresponsibility

⑤ I don't know

C13. Which of the following benefits of breastfeeding for infants were correct?

① Breastfeeding can lead to less babies sick

② Breast milk is the best natural food for babies

③ Infant formula is more nutritious than breast milk

④ Breastfeeding is conducive to the psychological development of infants

⑤ I don't know

C14. When keeping pesticides, attention should be paid to:

① Pesticides should be kept in a fixed, safe place

② Pesticides cannot be put together with food

③ If the hand is contaminated with pesticides, washing hand is not necessary as long as the skin is damaged.

④ Pesticide should be kept in the place where children can not touch

⑤ I don't know

C15. Out during thunderstorms, which of the following statements are correct?

① Hiding under the big tree

② Staying away from the high voltage line

③ Avoid using cell phone

④ Standing in the highlands

⑤ I don't know

D Situational questions (Please read the material first and then answer the questions. There is only one correct answer for a single choice question and two or more correct answers for a multiple choice question. If you do not know, single choice please choose ④, multiple choice please choose ⑤.)

BMI refers to body mass index (BMI), which is a commonly used international standard to measure body fat and thinness as well as health. This is calculated by dividing weight (kg) by height (m) squared, or BMI= weight/height^2 (kg /m^2). For Chinese adults, BMI<18.5 means underweight, 18.5≤BMI<24 means normal weight, 24≤BMI<28 means overweight, and BMI≥28 means obesity.

D01. Mr. Li, 45 years old, 170 centimeters in height, 160 pounds in weight, how should his BMI be calculated? (Single choice)

① (80)2 /170=37.6 ② 80/(1.7)2 =27.7 ③ 160/(1.7)2=55.4 ④ I don't know

D02. With reference to the body mass index of Chinese adults, Mr. Li belongs to: (Single choice)

① obesity ② normal weight ③ overweight ④ I don't know

D03. What can Mr. Li do to control his weight? (Multiple choice)

① Do not eat staple food

② Exercise for at least half an hour every day

③ Reduce oil intake

④ Only eat fruits and vegetables

⑤ I don't know

D04. Which of the following diseases is Mr. Li prone to? (Single choice)

① hypertension ② osteoporosis ③gastric ulcer ④ I don't know

Part 2：eHealth literacy

Here's a description of yourself and how much you agree with it.

Items	strongly disagree	disagree	uncertain	agree	strongly agree
1. I know what health resources are available on the Internet	1	2	3	4	5
2. I know where to find helpful health resources on the Internet	1	2	3	4	5

3. I know how to find helpful resources on the Internet	1	2	3	4	5
4. I know how to use the Internet to answer my questions about health	1	2	3	4	5
5. I know how to use the health information I find on the Internet to help me	1	2	3	4	5
6. I have the skills I need to evaluate the health resources I find on the Internet	1	2	3	4	5
7. I can tell high quality health resources from low quality health resources on the Internet	1	2	3	4	5
8. I feel confident in using information from the Internet to make health decisions	1	2	3	4	5

Part 3：COVID-19 coping behaviors

How often have you practiced the following behaviors in the past two weeks

Items	none	a small amount of time	sometimes	most of the time	almost all time
1. Reduce instances of going to public places	1	2	3	4	5
2. Wear a mask when going out	1	2	3	4	5
3. Cover your mouth and nose when you cough or sneeze	1	2	3	4	5
4. Maintain hand hygiene	1	2	3	4	5
5. Disinfect daily necessities	1	2	3	4	5
6. Open windows for ventilation to maintain air circulation	1	2	3	4	5
7. Follow the latest developments on COVID-19	1	2	3	4	5
8. Take body temperature frequently	1	2	3	4	5
9. Contact relatives and friends online	1	2	3	4	5
10. Communicate or confide with others when you are in trouble	1	2	3	4	5
11. Smok because of COVID-19	1	2	3	4	5
12. Drink alcohol because of COVID-19	1	2	3	4	5
13. Insist on physical exercise	1	2	3	4	5
14. Maintain adequate nutrition and balanced diet	1	2	3	4	5
15. Guarantee good sleep	1	2	3	4	5

Part 4：COVID-19 knowledge

1.What is the source of infection of COVID-19【multiple answers allowed】

① Seafood

② Wild animals

③ Patients with new coronavirus pneumonia

④ Patients with asymptomatic infection

⑤ I don't know

2.How long is the incubation period of COVID-19?【single answers allowed】

① 1-14 days

② 15-28 days

③ > 28 days

④I don't know

3.How does COVID-19 spread?【multiple answers allowed】

①Blood transfusions

②Respiratory droplets from infected people

③ Airborne transmission

④ Direct contact with infected people

⑤ Touching contaminated objects/ surfaces

⑥ Contact with contaminated animals

⑦ Eating contaminated food

⑧ Drinking unpurified/untreated water

⑨ I do not know

4. Who is prone to infection with COVID-19?【multiple answers allowed】

① Children

② Elderly people

③ Young people

④ Patients with chronic diseases

⑤ I don't know

5.What are the main symptoms of COVID-19?【multiple answers allowed】

①Fever

②Cough

③ Shortness of breath and breathing difficulties

④ Muscle pain

⑤ Headache

⑥ Diarrhea

⑦ No symptoms

⑧I don't know

Part 5：Sociodemographic characteristics

1. Your age: ________

2.Gender:

①Male

②Female

3.Your residence:

①Urban

②Rural

4. College year:

①Freshman

②Sophomore

③ Junior

④ Senior

5.Academic major:

①Medicine

②Others

6.Which level do you think your family's economic income corresponds to in the local area?

①High

②Medium

③ Low

7.How do you think your health status:

①Good

②Medium

③ Bad

8.Do your family member or friend infected with coronavirus?

①Yes

②No
