# Supplementary material for: Associations Between Health Literacy, eHealth Literacy, and COVID-19–Related Health Behaviors Among Chinese College Students: Cross-sectional Online Study
Source: J Med Internet Res. 2021 May 6;23(5):e25600. doi: 10.2196/25600 (PMC8104003; doi:10.2196/25600)
Supplement: Multimedia Appendix 2 [file jmir_v23i5e25600_app2.pdf]

Comparison of COVID-19-related health behaviors among college students with different demographic characteristics（$\bar{x} \pm s$）

**Variables**	**COVID-specific precautionary behaviors**	***P***	**Effect size**	**Conventional health behaviors**	***P***	**Effect size**	**COVID-19-related health behaviors**	***P***	**Effect size**
Gender		<.01	.212		.07	.085		.04	.096
Male	29.83±5.28			23.57±4.11			53.40±8.52		
Female	30.92±5.00			23.24±3.60			54.17±7.47		
Residence		.011	.118		.017	.109		.01	.130
Urban	30.68±5.17			23.64±3.99			54.33±8.03		
Rural	30.07±5.17			23.22±3.76			53.29±8.01		
College year		<.01	.059		<.01	.024		<.01	.049
Freshman	32.48±4.56			23.92±4.04			56.41±7.62		
Sophomore	30.62±5.21			24.01±3.76			54.63±7.91		
Junior	29.32±5.33			22.81±3.80			52.13±8.05		
Senior	29.11±4.76			22.75±3.76			51.86±7.66		
Academic major		.014	.114		.232	.054		.03	.100
Medicine	30.68±4.97			23.53±3.81			54.21±7.82		
Others	30.09±5.33			23.32±3.92			53.41±8.20		
Family economic level		<.01	.008		<.01	.015		<.01	.018
High	30.55±5.80			24.04±4.00			54.59±8.82		
Medium	30.50±4.75			23.26±3.73			53.76±7.46		
Low	28.95±5.39			22.43±4.05			51.38±8.38		
Self-reported health status		<.01	.022		<.01	.035		<.01	.029
Good	30.72±5.10			23.75±3.82			54.47±7.87		
Medium	29.01±5.05			22.20±3.54			42.81±7.54		
Bad	27.31±6.54			20.31±5.71			47.63±11.99		
Family member or friend infected with coronavirus		<.01	.859		<.01	.464		<.01	.777
Yes	34.59±3.39			25.14±5.22			59.73±7.80		
No	30.20±5.16			23.35±3.80			53.55±7.96		
Health literacy level		<.01	.516		<.01	.592		<.01	.624
Inadequate	29.28±5.31			22.50±3.76			51.77±7.91		
Adequate	31.87±4.58			24.70±3.66			56.56±7.35		
