# Supplementary material for: Associations Between Health Literacy, eHealth Literacy, and COVID-19–Related Health Behaviors Among Chinese College Students: Cross-sectional Online Study
Source: J Med Internet Res. 2021 May 6;23(5):e25600. doi: 10.2196/25600 (PMC8104003; doi:10.2196/25600)
Supplement: Multimedia Appendix 3 [file jmir_v23i5e25600_app3.pdf]

Factors associated with COVID-19-related health behaviors in Chinese college students.

Characteristic	COVID-specific precautionary behaviors									Conventional health behaviors								
	Model 1			Model 2			Model 3			Model 4			Model 5			Model 6		
	β	*t* test (*df*)[a]	*P* value	*β*	*t* test (*df*)[a]	*P* value	*β*	*t* test (*df*)[a]	*P* value	*β*	*t* test (*df*)[a]	*P* value	*β*	*t* test (*df*)[a]	*P* value	*β*	*t* test (*df*)[a]	*P* value
**Block 1: sociodemographic characteristics**																		
**Gender**																		
Male	Reference	N/A[b]	N/A	Reference	N/A	N/A	Reference	N/A	N/A	Reference	N/A[b]	N/A	Reference	N/A	N/A	Reference	N/A	N/A
Female	.110	5.044(12)	***<.001***[c]	.142	6.581(12)	***<.001***	.153	7.671(12)	***<.001***	-.050	-2.140(12)	***.032***	-.011	-.476(12)	.634	-.003	-.119(12)	.905
**Residence**																		
Urban	.030	1.337(12)	.181	.027	1.211(12)	.226	.001	.030(12)	.976	.017	.716(12)	.474	.013	.553(12)	.580	-.007	-.306(12)	.760
Rural	Reference	N/A[b]	N/A	Reference	N/A	N/A	Reference	N/A	N/A	Reference	N/A[b]	N/A	Reference	N/A	N/A	Reference	N/A	N/A
**College year**																		
Freshman	.185	6.655(12)	***<.001***	.155	5.678(12)	***<.001***	.094	3.707(12)	***<.001***	.071	2.424(12)	***.015***	.034	1.188(12)	.235	-.012	-.419(12)	.676
Sophomore	.035	1.185(12)	.236	.024	.825(12)	.409	-.017	-.657(12)	.511	.119	3.827(12)	***<.001***	.105	3.504(12)	<.001	.074	2.566(12)	***.010***
Junior	-.048	-1.680(12)	.093	-.044	-1.588(12)	.113	-.061	-2.384(12)	***.017***	-.009	-.300(12)	.764	-.004	-.150(12)	.881	-.017	-.614(12)	.540
Senior	Reference	N/A[b]	N/A	Reference	N/A	N/A	Reference	N/A	N/A	Reference	N/A[b]	N/A	Reference	N/A	N/A	Reference	N/A	N/A
**Academic major**																		
Medicine	.004	.181(12)	.857	-.018	-.834(12)	.404	-.029	-1.470(12)	.142	.002	.100(12)	.920	-.024	-1.098(12)	.272	-.032	-1.531(12)	.126
Others	Reference	N/A[b]	N/A	Reference	N/A	N/A	Reference	N/A	N/A	Reference	N/A[b]	N/A	Reference	N/A	N/A	Reference	N/A	N/A
**Family economic level**																		
High	.033	.887(12)	.375	-.014	-.375(12)	.708	.039	1.144(12)	.253	.101	2.529(12)	***.012***	.042	1.093(12)	.274	.082	2.198(12)	***.03***
Medium	.089	2.474(12)	***.013***	.073	2.075(12)	***.038***	.122	3.753(12)	***<.001***	.060	1.590(12)	.112	.040	1.102(12)	.271	.077	2.183(12)	***.03***
Low	Reference	N/A[b]	N/A	Reference	N/A	N/A	Reference	N/A	N/A	Reference	N/A[b]	N/A	Reference	N/A	N/A	Reference	N/A	N/A
**Self-reported health status**																		
Good	.266	4.072(12)	***<.001***	.259	4.075(12)	***<.001***	.205	3.483(12)	***.001***	.322	4.654(12)	***<.001***	.314	4.702(12)	***<.001***	.273	4.247(12)	***<.001***
Medium	.139	2.129(12)	***.033***	.139	2.193(12)	***.028***	.114	1.944(12)	.052	.189	2.739(12)	***.006***	.189	2.848(12)	***.004***	.171	2.665(12)	***.008***
Bad	Reference	N/A[b]	N/A	Reference	N/A	N/A	Reference	N/A	N/A	Reference	N/A[b]	N/A	Reference	N/A	N/A	Reference	N/A	N/A
**Family member or friend infected with coronavirus**																		
Yes	.141	6.604(12)	***<.001***	.120	5.759(12)	***<.001***	.095	4.917(12)	***<.001***	.065	2.893(12)	***.004***	.040	1.822(12)	.069	.021	.992(12)	.321
No	Reference	N/A[b]	N/A	Reference	N/A	N/A	Reference	N/A	N/A	Reference	N/A[b]	N/A	Reference	N/A	N/A	Reference	N/A	N/A
**Self-reported coronavirus knowledge level**	.257	12.063(12)	***<.001***	.221	10.493(12)	***<.001***	.156	7.875(12)	***<.001***	.123	5.458(12)	***<.001***	.079	3.561(12)	***<.001***	.030	1.373(12)	.170
**Block 2: Health literacy**																		
Health **literacy**	—[d]	—	—	.221	10.027(1)	***<.001***	.149	7.175(1)	***<.001***	—	—	—	.273	11.827(1)	***<.001***	.219	9.674(1)	***<.001***
**Block 3: eHealth literacy**																		
eHealth **literacy**	—	—	—	—	—	—	.368	17.987(1)	***<.001***	—	—	—	—	—	—	.277	12.370(1)	***<.001***
R^2	.179			.222			.337			.080			.144			.209		
Adjusted R^2	.174			.216			.332			.074			.138			.203		
R^2 Change	—			.042			.115			—			.064			.065		
F value	33.890			40.692			67.449			13.393			24.045			35.083		
F value Change	—			100.546			323.518			—			139.878			153.008		
P value	*<.001*			*<.001*			*<.001*			*<.001*			*<.001*			*<.001*		

[a] Two-tailed t tests were performed.

[b] N/A: not applicable

[c] Italics and bold indicate statistical significance between groups.

[d] —: Data not included in this model.
